# Synthesis and application of colloidal CdS quantum dots as interface modification material in perovskite solar cells

**DOI:** 10.3906/kim-2107-2

**Published:** 2021-09-16

**Authors:** Esma YENEL

**Affiliations:** Department of Electricity and Energy, Konya Technical University, School of Technical Science, Konya Turkey

**Keywords:** Colloidal CdS quantum dots, perovskite solar cells

## Abstract

In this study, colloidal CdS quantum dots were synthesized, structurally characterized, and their effect on performance of perovskite solar cells was observed by using them as interface modification agent between TiO_2_/perovskite. Colloidal CdS quantum dots were synthesized based on two-phase method and characterized by X-ray diffraction and Transmission Electron Microscopy techniques. The average particle size of CdS quantum dots have found to be around 5 nm. Oleic acid was used as capping agent during synthesis to lead solubility in organic solvents. Obtained quantum dots are coated on compact TiO_2_ layer for surface modification. A decrease was observed when oleic acid capped CdS quantum dots were used at interface, while significant improvement was observed when ligand exchange was carried out by pyridine before perovskite layer. Reference solar cells showed 11.6% efficiency, while pyridine capped CdS modified solar cells’ efficiency was 13.2%. Besides the improvement in efficiency, reproducibility of solar cells also was increased by using pyridine capped CdS as interface material.

## 1. Introduction

Recently, due to their low cost, high efficiency, and facile fabrication, perovskite solar cells have become more attractive for many researchers. Since Miyasaka and co-workers firstly reported in 2009, perovskite solar cell (PSC) technology has undergone an improvement from 3.8% to around 25% [1,2]. A basic perovskite solar cell consists of a transparent conductive layer such as Florine doped Tin Oxide (FTO) or Indium doped Tin Oxide (ITO), an electron transport layer, light sensitive perovskite layer, a hole transport layer, and finally a metal electrode. As it is valid for all layers, electron transport layer plays an important role for high efficiency in PSCs. TiO_2_ is one of the most used electron transport layer due to its’ various fabrication method such as spin coating, spray coating, sputter, etc. [3–5]. Independent from preparation technique, TiO_2_ structure includes some problems such as oxygen vacancies and nonstoichiometric defects especially located on TiO_2_ surface [6,7]. Those defects prevent electron flow that results in bad performance of perovskite solar cells. Some of researchers reported some different materials such as SnO_2_, ZnO, CdS and WOx instead of TiO_2_ as electron transport layer [8–11]. Although CdS as an electron transport layer is still far from satisfactory, it may be an excellent interface material for modification and passivation of TiO_2_ surface. Recently, Hwang et al. reported that CdS, as a modification material for mesoporous TiO_2_ layer, lead improvement in stability of perovskite solar cells [12]. Zhao et al. used CdS as an additive to precursor solution and observed significant reduction in recombination[13]. Dong et al. used CdS as electron transport layer and observed 16.5% efficiency in PSCs [14]. Wessendorf et al. observed a decrease in hysteresis by using CdS as electron transport layer [15]. Cd diffusion through to perovskite layer leads an increase in grain size resulting better efficiency [16]. Mohamadkhania et al. used CdS on SnO_2_ surface as interface modifier and observed decrease in hysteresis and increase in efficiency [17]. Ma et al. showed that chemically deposited CdS on TiO_2_ surface improves the efficiency from 10.31% to 14.26% [18].

In this work, different from the other works previously reported, we used colloidal CdS quantum dots for modification of TiO_2_ surface. Firstly, we synthesized oleic acid capped CdS and directly used those materials as interface agent. For comparison, we carried out ligand exchange procedure with pyridine to obtain pyridine capped CdS. Pyridine is a small and electron-rich molecules that it contributes electron transfer in such devices. Some researchers used pyridine and similar small molecules between electron transport layer and perovskite layer [19]. In addition, pyridine can be easily replaced with oleic acid without further chemical process. So, pyridine would be one of the best choices to eliminate oleic acids. Both types of CdS quantum dots were used at TiO_2_/perovskite interface, and the results were compared with nonmodified PSCs.

## 2. Materials and methods

### 2.1. Synthesis of colloidal CdS quantum dots

In the synthesis of quantum dots, the two-phase method has been used. Quantum dots are obtained at the interface between the organic phase and the water phase. First, the sulphur source thiourea, which is used as a slow and controlled release agent by hydrolysis on the surface of cadmium myristate particles not fully dissolved in the water phase, is preferred. In the reaction medium to be created in this way, depending on the time and concentration, the particle grows very slowly and in a controlled manner at the interface. The applied synthesis method is given below.

Cadmium myristate particles start crystal formation on the surface, and then the growth of crystals was achieved at the interface. In the reaction added sulphur source thiourea, by controlled hydrolysis, provides sulphur to the environment. CdS cores will be created. The resulting structure is transferred from the interface to the organic phase with the help of the surfactant in toluene after the addition of the toluene phase. During this transition, growth will continue at the interface.

Separating the reacted parts of cadmium myristate from the surface of particles, the remaining cadmium myristate reacts rapidly with sulphur to form the same system. Particle formation will continue over it. 0.4 g cadmium myristate is suspended in 80 mL of water under argon at 100 °C for 15 min. At this stage, the solution of 0.060 g thiourea in 5 mL of water should be added to the medium by syringe. Immediately after, 80 mL of toluene containing 1 mL of oleic acid is added and continued to stir at 100 °C. A total of 2 mL of sampling is done every 30 min from toluene phase, and growth control of crystal with the aid of UV and fluorescence is done. At the end of the reaction, the quantum dots are removed from the organic phase with the help of ethanol separated by precipitation and stored for analysis. Samples taken during the reaction are also precipitated with ethanol and stored. Coated with surfactant materials such as electron-poor oleic acid, TOPO electron flow is low in solar cells produced with quantum dots. In cells produced with quantum dots coated with surface-active materials such as thiophene and pyridine, which are rich in electrons, electron flow is high due to conjugated bonds. Therefore, during the studies, electron-rich surfactants such as pyridine materials have been used.

### 2.2. Fabrication of perovskite solar cells

#### 2.2.1. Preparation of reference perovskite solar cells

Perovskite solar cells (PSCs) were fabricated on FTO coated glass. Fluorine doped tin oxide (FTO) coated glass substrates were cut into 1.5 cm×1.5 cm square pieces. FTO glass was ultrasonically cleaned with hellmanex, de-ionized water, acetone, and isopropyl alcohol for 10 min and dried with nitrogen. Before coating, oxygen plasma treatment was carried out to remove organic impurities and to activate the surface. Compact TiO_2_ layer was coated by spray coating technique. TiO_2_ solution was prepared by dissolving 3 mL of titanium isopropoxide in absolute ethanol. Another solution with 2 mL of acetylacetone in 6 mL of absolute ethanol was prepared. Acetylacetone solution was added to stirring titanium isopropoxide dropwise. The mixture was kept overnight by stirring at room temperature. Before spray coating, stock solution was 1:1 diluted with absolute ethanol. FTO glasses were heated to 450 °C and coated by spray then kept for 3 min at the same temperature and cooled down to room temperature slowly.

Perovskite in DMSO:DMF solvent was prepared according to previously published procedure Zhang et al [20]. The perovskite precursors, consisting of 922 mg PbI_2_ and 349.8 mg MAI were dissolved in 900 μL DMF and 100 μL DMSO. Then, perovskite layer was deposited statically by spin-coating at 6000 rpm for 30 s. A total of 100 μL of sec butyl alcohol as antisolvent was dropped at 7th s during spinning. At the second step, sec-butyl alcohol was added statically on perovskite coated layer and kept for 12 s and spin coated at 6000 rpm for 30 s. Final perovskite films were dried at 100 °C for 30 min.

Spiro-OMeTAD solution consisting of 65 mg of spiro-OMeTAD, 20 μL of 4-tert-butyl pyridine, 70 μL of Li-TFSI (170mg mL-1 in acetonitrile), and 1mL of chlorobenzene was prepared and spin-coated at 4000 rpm for 30 s onto perovskite layer.

Finally, 100 nm of Au was thermally evaporated onto HTM layer by using a shadow mask. Active area of each electrode was calculated to be 0.023 cm^2^.

#### 2.2.2. Preparation of oleic acid and pyridine capped perovskite solar cells

For OA-CdS modified perovskite solar cells, oleic acid capped quantum dots dissolved in chlorobenzene (5 mg/mL) were spin casted onto TiO_2_ surface at 5000 rpm for 40s and dried at 100 °C for 5 min. Then, perovskite solution was spin casted as explained in text above. PYR-CdS modified solar cells were prepared by pyridine washing after coating OA capped CdS. For this concept, after coating OA capped CdS on TiO_2_ surface, 70 ul pyridine was dropped on film during spinning at 5000 rpm and then dried at 100 °C 5 min. After pyridine treatment, perovskite layer was deposited as explained above.

#### 2.2.3. Characterizations of perovskite solar cells

IV characterizations were carried out in nitrogen filled glove box system under AM 1.5 solar simulator by Keithley 2400 power source. Light intensity was measured with a calibrated KIPP&ZONEN pyronometer and calculated to be 80 mW/cm2

## 3. Results and discussion

**Figure 1:**
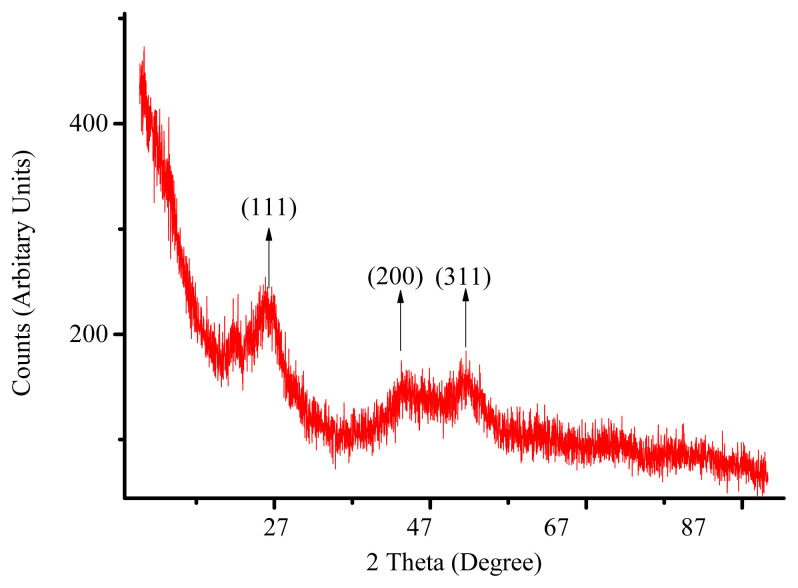
XRD spectra of colloidal CdS quantum dots.

**Figure 2:**
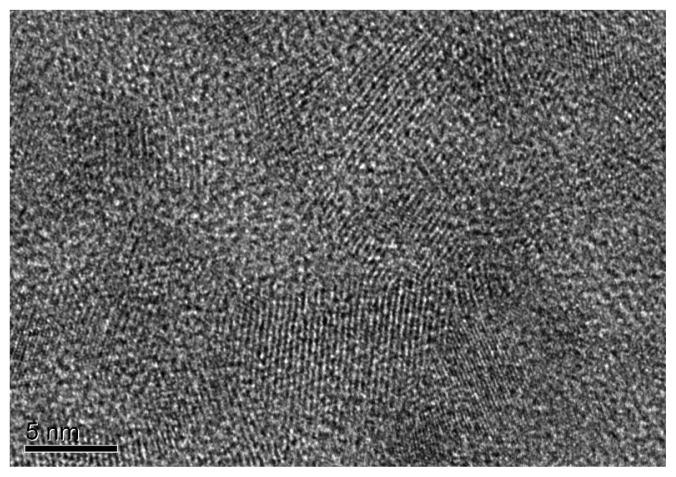
TEM pictures of CdS quantum dots.

The XRD spectrum of CdS quantum dots showed that the crystal structure of CdS QDs is cubic with an excellent match to XRD spectrum of cubic bulk CdS (JCPDS no. 10-0454). XRD spectrum of CdS quantum dots had 3 peaks at 26.6° (corresponds to 111 plane of cubic CdS), 44.11° (corresponds to 200 plane of cubic CdS), and 52.26° (corresponds to 311 plane of cubic CdS). These 3 peaks are typical 2θ values for cubic CdS crystal (JCPDS no. 10-0454). Also, the broadening in these peaks was a proof of quantum dot formation. By applying Scherrer equation, the size of CdS quantum dots were found to be 5 nm (see Figure 1). Transmission electron microscopy images also supports the formation of CdS quantum dots (see Figure 2). It is clear from the Figure 1 that average particle size of quantum dots is approximately 5 nm, and a homogenous particle size distribution is observed.

**Figure 3:**
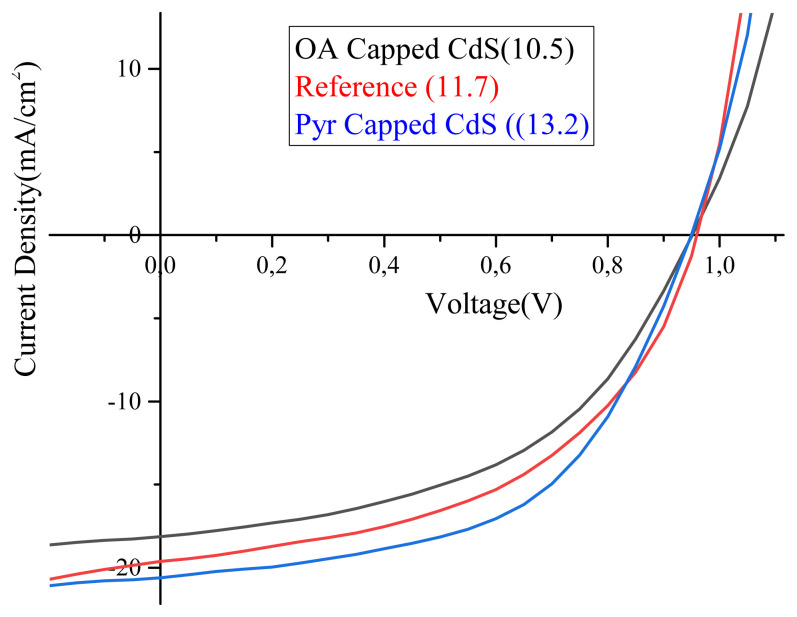
IV characterizations of perovskite solar cells.

Synthesized quantum dots are used as interface layer between TiO_2_ and perovskite layer in forms of oleic acid capped and pyridine capped. Figure 3 shows IV characterizations of reference and CdS included perovskite solar cell. As it is clear from Figure 3 and Table, oleic acid capped CdS lead a decrease from 11.7% to 10.5% in PSCs’ performance. This is most probably due to long hydrocarbon chain of the capping agent the prevent electron injection in such devices. Oleic acid has 18 carbons, which means that it takes too long for hopping process of electrons from one side to other side. However, in case pyridine capped CdS, performance of PSCs is obviously increased from 11.7% to 13.2%. At first, this improvement may be attributed to UV absorption of CdS and its’ contribution to electron transfer process. If this comment was true, we should have observed improvement for both OA and pyridine capped CdS. However, pyridine capped CdS show improvement, while it is vice versa for OA capped. The role of OA is discussed above. So, we may suggest that the absorption of CdS does not influence the electron transfer, therefore, efficiency. This improvement may be attributed to two reasons. The first one may be the basic explanation that electron rich structure and molecular volume of pyridine facilitate electron transfer from perovskite to TiO_2_. Pyridine, due to its’ molecular structure, passivates the surface states of CdS quantum dots; besides, it provides an electron rich media that facilate electron hopping process. The second one can be attributed to the coordination of excess amount of pyridine to the uncoordinated Pb^+2^ ions that increases crystal quality of perovskite, which results in better performance of PSCs. As well known in literature, uncoordinated Pb^2+^ in perovskite layer immigrates through to HTM layer and this movement dramatically decrease stability and efficiency of PSCs [21,22]. Xue et al. reported that amino and hydroxy groups facilitate crystal formation of perovskite and coordinate to the uncoordinated Pb^2+^ [23]. Similar process may occur in case pyridine existence at TiO_2_/perovskite interface.

Table summarized the performance parameters of PSCs. In all cases, Voc values are observed to be 950 mV, while Isc values varies from 18.14 to 20.6 depending on efficiency. As explained above, observation of low current in OA capped CdS is most probably due to long hydrocarbon chain of oleic acid side. On the other hand, better current values are observed in case pyridine capped CdS that support our attributions. Beside Isc values, FF values are also improved by pyridine capped CdS included PSCs.

**Figure 4:**
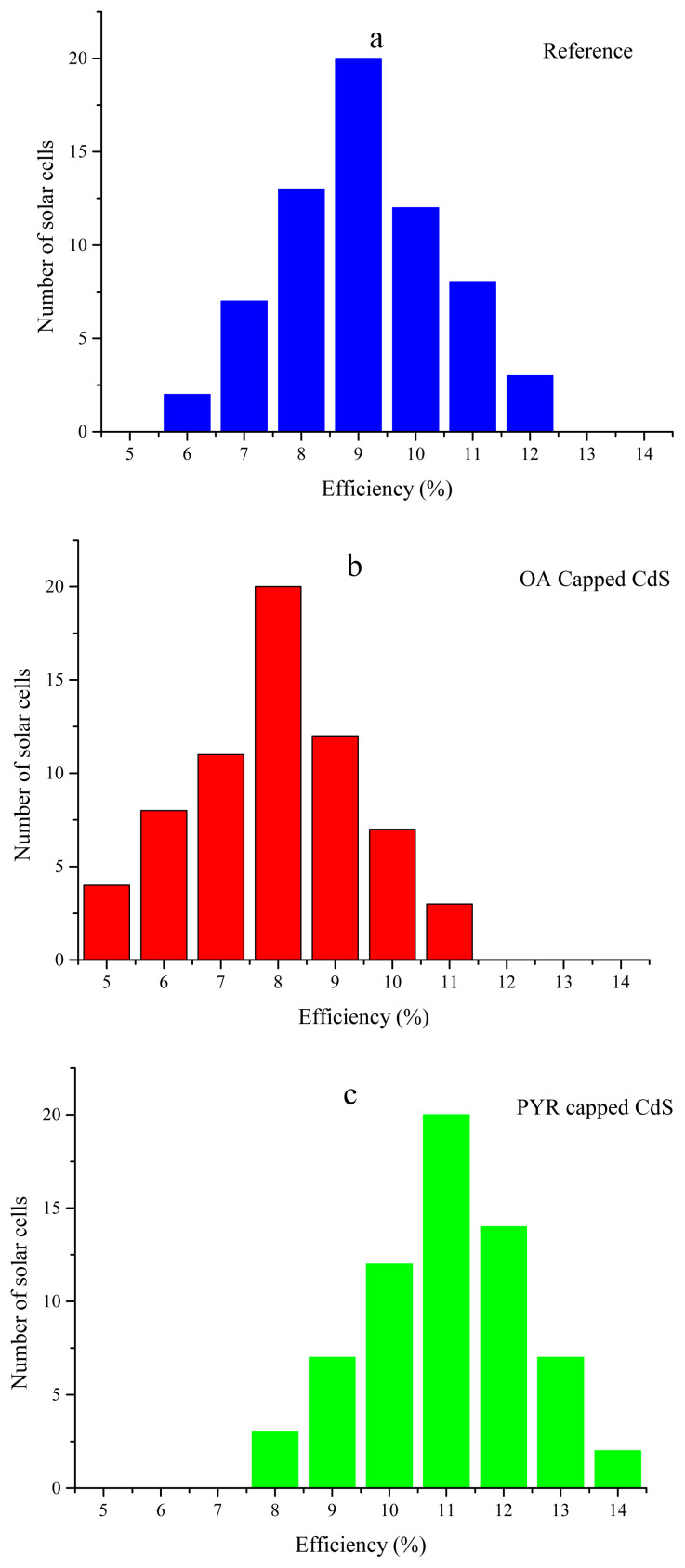
Reproducibility results of PSCs. (a) refers to reference, (b) refers to oleic acid capped CdS layer included, and (c) refers to pyridine capped CdS included PSCs.

Reproducibility is another important parameter for PSCs. Reproducibility can be considered an indicator for crystal quality of perovskite. Figures 4a–4c show average efficiency values of a number of PSCs. It is clear from Figures 4a–4c that pyridine capped CdS significantly improves reproducibility of PSCs. Pyridine capped CdS included PSCs show narrower efficiency distribution than reference and OA capped CdS included PSCs. As discussed above, pyridine passivates CdS surface and fill electron traps as well as coordinates to uncoordinated Pb^2+^, which increases crystal quality of perovskite layer.

## 4. Conclusion

In this work, we focused on TiO_2_ and perovskite layer interface modification for better efficiency and reproducibility. The results showed that not only quantum dot type but also capping agent play an important role on efficiency and reproducibility of PSCs. Especially electron rich groups facilitate electron transfer from perovskite to TiO_2_ layer and increase crystal quality of perovskite by interaction with uncoordinated Pb^2+^ cations, which increases efficiency and reproducibility.

## Figures and Tables

**Table. IV:** data of PSCs.

Concept	Efficiency	Isc	Voc	FF
Reference	11.7	19.65	950	0.50
CdS OA	10.5	18.14	950	0.49
CdS Pyr	13.2	20.6	950	0.54
